# Transcriptomic signature of differentiating catshark cartilage unravels the co-evolution of the Spp2 gene family with skeletal mineralisation in cartilaginous fish

**DOI:** 10.1186/s12915-026-02593-9

**Published:** 2026-04-11

**Authors:** Mélanie Debiais-Thibaud, Nicolas Leurs, Virginia Panara, Nathanaëlle Saclier, Elise Gueret, Ronan Lagadec, Marc Fichter, Nina Besset, Théo Deremarque, Camille Martinand-Mari

**Affiliations:** 1 Institut des Sciences de l’Évolution de Montpellier, Université de Montpellier, CNRS, IRD, Montpellier, 34090 France; 2 Department of Organismal Biology, Uppsala University, Uppsala, 75236 Sweden; 3 MGX- Montpellier GenomiX, Univ. Montpellier, CNRS, INSERM, Montpellier, France; 4 Sorbonne Université, CNRS, Biologie Intégrative des Organismes Marins, BIOM, Banyuls-sur-Mer, France

**Keywords:** Biomineralisation, Secreted phosphoprotein spp2, Elasmobranch, Mineralised cartilage

## Abstract

**Background:**

Skeletal mineralisation, achieved through the controlled precipitation of calcium phosphate, represents a major evolutionary innovation in early vertebrates. While this process has been extensively studied in bony species, it remains poorly understood in cartilaginous fishes, which lack endochondral bone. In this study, the small-spotted catshark (*Scyliorhinus canicula*) was used as a model to investigate the genetic basis of cartilage differentiation and subsequent mineralisation.

**Results:**

Two stages in late embryogenesis were examined: one preceding and one following the onset of vertebral mineralisation. Using a bulk RNA sequencing approach, we identified a set of genes characterising the early differentiation of chondrocytes and cartilage synthesis, shared among jawed vertebrates. Strikingly, only a very limited number of genes previously identified in mammalian skeletogenesis showed upregulated expression in the catshark mineralising chondrocytes. Among these, particular attention was given to the *spp2* (secreted phosphoprotein 2) gene family. Unlike in bony vertebrates, where *spp2* exists as a single and rather little-studied gene, cartilaginous fishes possess multiple gene duplicates with contrasted sites of expression. Through phylogenetic analyses and gene expression studies in the catshark, we showed how, despite the presence of shared molecular components likely inherited from their last jawed vertebrate ancestor, cartilaginous and bony fishes have independently evolved distinct cartilage mineralisation strategies.

**Conclusions:**

This work highlights the diversity of skeletal development mechanisms and underscores the importance of cartilaginous fishes in broadening our understanding of how vertebrate mineralisation evolved. By contrasting conserved and lineage-specific features, the study provides new insights into the independent trajectories that shaped the skeletal structures of modern vertebrates.

**Supplementary Information:**

The online version contains supplementary material available at 10.1186/s12915-026-02593-9.

## Background

Early vertebrates have evolved a diversity of bio-mineralised tissues such as dentine, enameloid, bone and mineralised cartilage more than 450 million years ago, by the evolution of the controlled deposition of calcium phosphate crystals in the extracellular matrix of various tissues [1–4]. Extant cartilaginous fishes (sharks, rays, skates and chimaeras) have secondarily lost the ancestral ‘dermal’ or ‘perichondral’ bone of early vertebrates, but show a skeleton mostly built from partially mineralised cartilage (reviewed in [5]). Their peculiar mineralised tessellated cartilage and more extreme skeletal features described as ‘bone-like’ tissues [6–11] are of particular interest regarding the origin and diversification of a mineralised skeleton in vertebrates. The skeletal cell diversity of cartilaginous fishes has poorly been studied from a developmental genetic perspective, due to limited molecular data until recently. As a result, the molecular signature of skeletal development in cartilaginous fishes has previously been partially uncovered by the study of candidate genes known to be involved in the function of tetrapod articular or hypertrophic chondrocytes, or of tetrapod osteocytes [12–15]. These studies highlighted the existence of different skeletal tissue types based on their histology, mineralisation ability and the expression of genes coding for several collagenous and non-collagenous components of the skeletal extracellular matrix [6, 16–21]. Gnathostome-ancestral aspects of skeletal development and mineralisation were uncovered (e.g. the involvement of type X collagen in all types of mineralised tissues [17]), as well as complex evolution in some gene families where paralogs evolved different roles following gene duplication (e.g. the bone-GLA and matrix-GLA protein family [18]). Several of these results pointed towards a gnathostome ancestral toolkit for extracellular mineralisation, despite the fact that the loss of bone in cartilaginous fishes was associated to genomic data in these species such as the absence of the *Osterix* transcription factor (*Sp7*), or genes encoding secreted calcium-binding phosphoproteins (SCPP) [14, 20, 22].

The recent publication of high-quality molecular (genomic and transcriptomic) datasets in cartilaginous fishes now opens more comprehensive perspectives to describe the molecular genetics of skeletal development and evolution [23–27]. To better characterise the putative gnathostome-ancestral mineralisation toolkit, we used a transcriptomic approach in the small-spotted catshark *Scyliorhinus canicula*, leaning on a recently assembled and thoroughly annotated genome and dense RNA sequencing (RNAseq) data from ontogenetic stages and adult tissues [27]. We identified skeletal genes with differential expression between two developmental stages covering the pre- and post-mineralisation periods of the skeleton [28]. Previously studied candidate genes were recovered from this analysis as well as a multi-paralog gene family, orthologous to the single *spp2* (*secreted phosphoprotein 2*) gene in bony fishes. Our results highlight the parallel evolution of secreted phospho-proteins involved in skeletal mineralisation in bony and cartilaginous fishes. The comparison between our data and data obtained from bony fish highlights a restricted set of ancestral genetic actors involved in skeletal development and mineralisation, and identifies derived characteristics of either cartilaginous or bony fishes leading to the diversification of the vertebrate skeleton.

## Results

### Transcriptomic signature of skeletal tissue differentiation during embryonic development

**Fig. 1:**
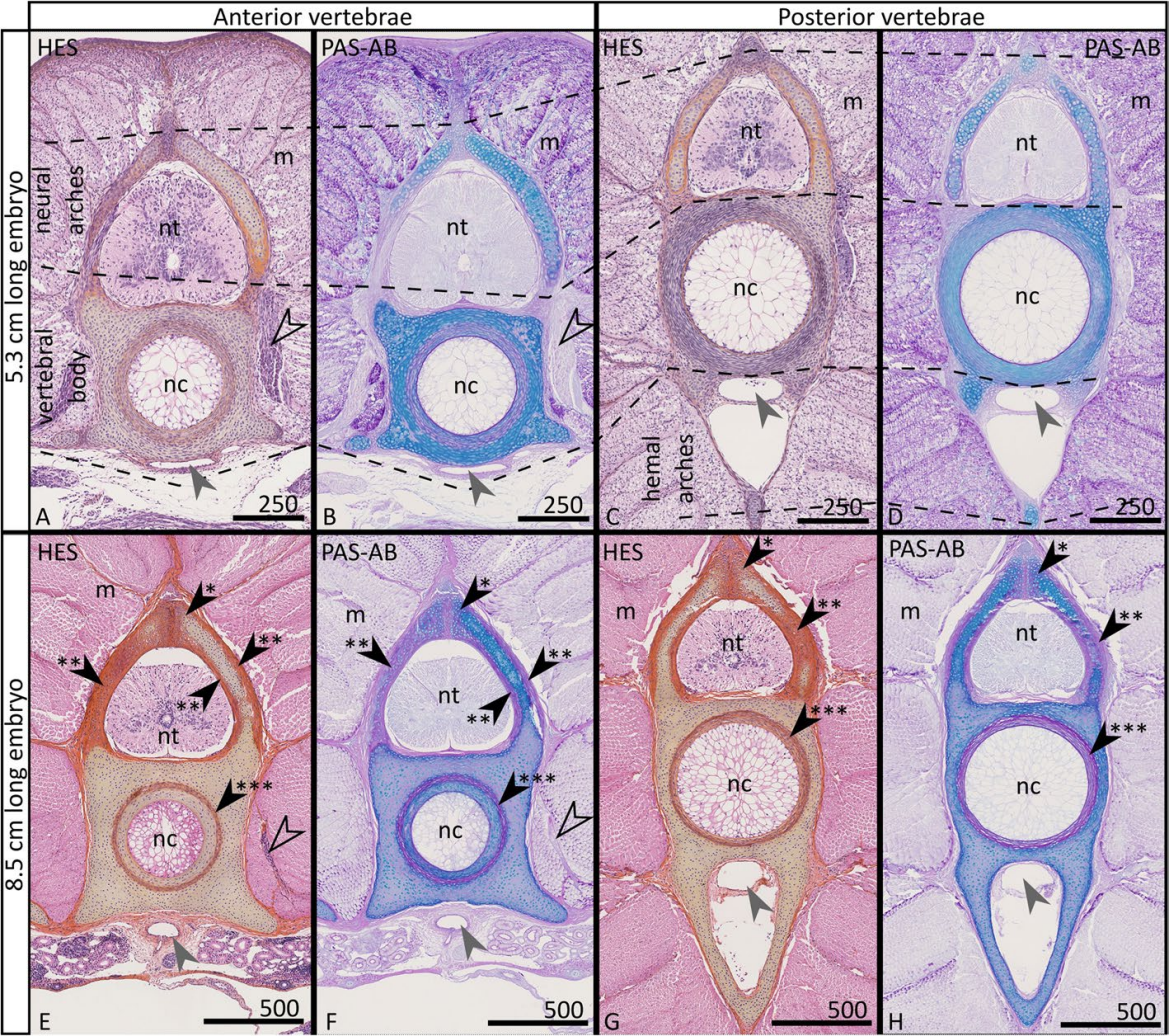
Transverse, 7-µm-thick, sections of anterior (**A**, **B**, **E**, **F**) and posterior (**C**, **D**, **G**, **H**) vertebrae of a 5.3-cm-long (**A**–**D**) and of an 8.5-cm-long (**E**–**H**) *Scyliorhinus canicula* embryo stained with haematoxylin–eosin-safran (HES) or with periodic acid Schiff-Alcian blue (PAS-AB). Mineralisation of the vertebral tissues occurs in the extracellular matrix of chondrocytes in the neural arches (black arrowhead with one asterisk), in the fibrous perichondrium covering the neural arches (black arrowhead with two asterisks), and in a fibrous and cartilaginous sheath in the vertebral body (black arrowhead with three asterisks). Neural arches of each vertebra enclose the neural tube (nt) from which nerves emerge (open arrowhead in **A**, **B**, **E**, **F**). Each vertebral body encloses the notochord (nc). Each vertebra is surrounded by abundant muscle tissue (m) and ventrally by vasculature (the dorsal aorta is shown with a grey arrowhead). Scale bars in microns

To identify genes associated with cartilage differentiation that would include the process of mineralisation, we sampled transverse sections in two zones of the vertebral column (‘anterior’, at the level of the pectoral fins; ‘posterior’, just posterior to the pelvic fins) of three individuals at two embryonic stages chosen before and after the time of vertebral mineralisation in the small-spotted catshark (~ 5 and 8 cm total length; [28]). Bulk RNA extraction and sequencing of these twelve samples were then performed in parallel (Fig. 1).

Variation of tissue proportions between samples being the most prominent issues in test of differential expression with bulk-RNA transcriptomics [29], we compared the abundance (as transcripts per million (TPM) values) of *col2a1* expression in the different libraries, considering this gene a marker of cartilage proportion in the sample (*col2a1* encodes the main collagen fibre produced by chondrocytes and is strongly expressed by chondrocytes at both stages chosen for the RNAseq sampling [6]. The abundance of *col2a1* transcript was variable between libraries (in a 1 to 10 scale, see XM_038786372.1 in Additional file 1: Table S1). We therefore generated a ‘col2a1 normalised’ count dataset in order to minimise variations in expression due to variations in cartilage volume between samples, hypothesising that chondrocyte expression of *col2a1* was comparable in single chondrocytes of younger and older embryos [30].

**Fig. 2:**
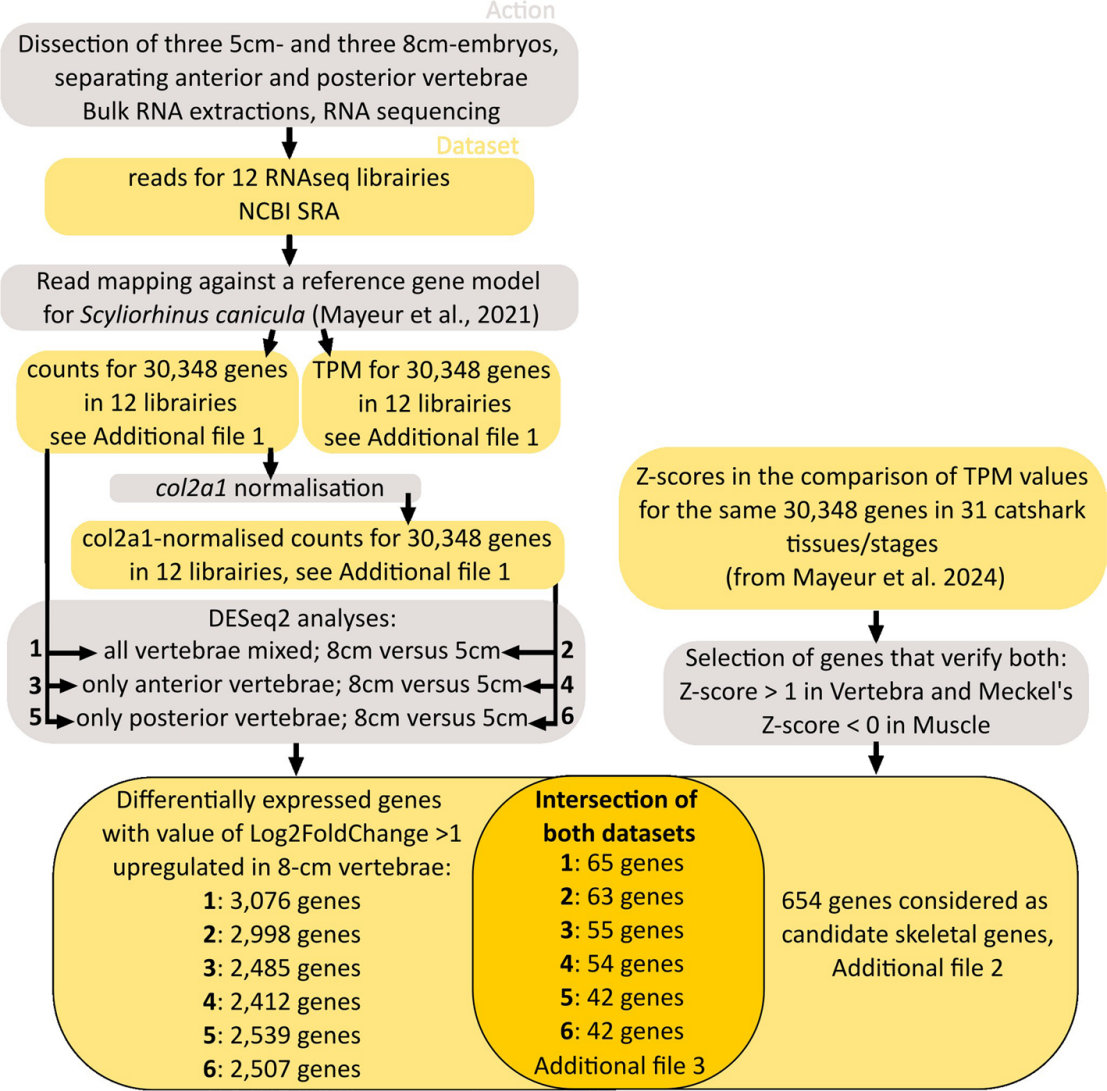
Graphical pipeline of successive actions performed (grey) and datasets obtained (orange) to identify skeletal genes differentially expressed in the time points of vertebral mineralisation

Differential expression analysis (DEseq) was performed on the original and ‘*col2a1* normalised’ datasets by comparing the abundance of each gene expressed in older embryos (8 cm long) relative to young embryos (5 cm long). This analysis retrieved 3076 genes upregulated at the older stage (Log2FC ≥ 1; padj < 0.05) from the original dataset, and 2998 upregulated genes from the ‘*col2a1* normalised’ dataset (2972 being common to both lists) in older embryos. The very similar results obtained with and without *col2a1* normalisation suggested that the analyses of triplicates were mostly robust to the variation observed in the proportion of cartilage. In the following, we further analyse only the results obtained from the *col2a1-*normalised dataset (Fig. 2).

Gene Ontology term (GO term) overrepresentation tests of these upregulated genes revealed strong enrichment in biological functions linked to muscle or nerve tissue development (Additional file 2: Tables S5–S6), suggesting a major participation of the tissues surrounding the vertebrae, such as muscle, vascular or nervous tissues (Fig. 1). To bypass this issue, we added a filtering step by generating a reference dataset for ‘skeletal genes’. To do so, we used a bulk RNAseq dataset obtained on thirty-one tissues that include vertebrae, Meckel’s cartilage, and muscle [27]. We selected genes with expression positively biased towards skeletal tissues (*Z*-score ≥ 1 in the Vertebrae and Meckel’s cartilage libraries) and negatively biased in our main ‘contaminating’ tissue (muscle: *Z*-score ≤ 0). This analysis produced a list of 654 transcripts, 398 of which were annotated in the catshark genome (Additional file 2: Table S7), with GO term overrepresentation pointing to biological processes linked to cartilage and bone development (Additional file 2: Table S8). The intersections between the DEseq and the skeletal candidate genes (Fig. 2) led to a reduced list of 65, 63, 54 and 42 genes when based on the original dataset, the col2a1-normalised dataset (all vertebrae combined), the col2a1-normalised anterior vertebra dataset and the col2a1-normalised posterior vertebra dataset, respectively (see Additional file 3: Table S9).

Many of these resulting genes encoded for non-coding RNAs or for predicted protein sequences that had no similarity to any vertebrate protein. This was often the case for ‘Cluster-’ sequences, transcripts produced by a de novo transcriptome assembly that show no alignment to the current reference genome in the catshark: these sequences are often of poorer quality as they cannot be cleared from assembly artefacts [27, 31]. As a consequence, these were not analysed further. In the list of the remaining 37 genes that were significantly upregulated in at least two of the three col2a1-normalised dataset analyses (Table 1), we also discarded seven genes with very low levels of expression (TPM < 15) in embryonic vertebrae (*yrdc*, *ptgir*, *spam1*, *skida1*, *cycs*,* gpr54*,* lrrc3*) and three genes annotated with function in autophagy or apoptosis (*atg2b*, *bcl6* and *smcr8a*). In the 27 remaining genes in the list, two were previously studied in skeletal development of the catshark with *bgp* shown to be expressed in perichondral cells surrounding mineralising cartilage (XM_038783991.1, [18]) and *scpp* shown to be expressed in mineralising teeth and scales (XM_038792863.1, [20]). To identify the cells involved in expression of the other upregulated genes, we performed in situ hybridisation for those with higher expression levels in both our embryonic RNAseq and the adult RNAseq data (TPM > 100) ([31]; Table 1). Of the thirteen genes studied, eight were significantly upregulated in the anterior vertebrae but not in the posterior samples. This result could be related to the observations reported by [28], according to which vertebral mineralisation is a gradual process that begins earlier in the thoracic vertebrae than in the post-pelvic region.

**Table 1:** Thirty-seven genes upregulated in 8 cm vertebrae, listed according to their TPM value in the vertebrae of 8 cm embryos. Significantly differentially expressed genes in 8-cm *versus* 5-cm-long embryo vertebrae (log2FC > 1 in different analyses see Additional file 3: Table S9). Genes in bold were selected for in situ hybridisation analysis. Expression level of each gene is given as TPM values in different samples. Embryonic stages: mean of the *col2a1*-normalised TPM values of anterior and posterior vertebra; adult skeletal tissues: TPM values in spinal chord, dental lamina, vertebrae, chondrocranium, Meckel’s (values with *Z*-score > 1 are in bold, TPM > 100 are italicised; see [27])

		**Log2 (fold change) > 1**					**TPM**							
**gene_ID**	**Annotation/best hit**	**Analysis 2**	**Analysis 4 (VA only)**	**Analysis 6 (VP only)**	**In situ test**	**Full name and function of human homolog**	**5 cm embryo**	**8 cm embryo**	**Spinal chord**	**Denticles**	**Dental lamina**	**Meckel.s**	**Vertebrae**	**Chondrocranium**
XM_038816264.1	**Otos**	1.14	1.18		Chondrocytes	Otospiralin; survival of the neurosensory epithelium of the inner ear	489	1504	4	3	3	***472***	***424***	***164***
XM_038787500.1	**CSTL1**	1.78	1.88	1.62	Spinal cord	Cystatin-like	321	1462	1665	1285	1068	***5701***	***5904***	***5294***
XM_038790375.1	**SPP2.3s2**	1.64	2.39		Mineralising chondrocytes	Secreted phosphoprotein 24-like	266	1205	1	0	1	***2692***	***2378***	***877***
XM_038788185.1	**SPP2.3s7**	3.83	4.17	2.40	Mineralising chondrocytes	Secreted phosphoprotein 24-like	33	774	1	0	1	***2708***	***2678***	***1067***
XM_038812782.1	RPP21	1.20	1.12	1.18		Ribonuclease P21 subunit	242	684	49	44	46	**71**	**72**	55
XM_038788196.1	**SPP2.3s5**	1.56	2.28		Mineralising chondrocytes	Secreted phosphoprotein 24-like	123	581	3	0	0	***725***	***854***	251
XM_038783991.1	**BGP**	2.05	2.20	1.79	Leurs et al., 2021	Bone gamma-carboxyglutamate protein	102	535	70	46	16	***340***	***723***	193
XM_038796173.1	**CTGF**	1.90	2.15	1.68	Spinal cord	CCN family member 2-like; chondrocyte proliferation and differentiation	92	430	19	26	27	***3055***	***1172***	***1845***
XM_038822098.1	btbd6	1.85	1.66	1.96	Spinal cord	BTB (POZ) domain containing 6b; involved in late neuronal development	94	425	**72**	30	47	**50**	**52**	**55**
Cluster-4003.0	Jph2	2.00	1.74	2.09		junctophilin 2 (cardiomyocyte)	65	376	**31**	10	9	**43**	**39**	**31**
XM_038806248.1	**clec3a**	1.14	1.30		Chondrocytes; intervertebral	C-type lectin domain family 3, member A; cartilage-derived C-type lectin	132	358	6	3	4	***414***	***738***	***537***
XM_038815745.1	rxrba	1.19	1.09	1.23		Retinoic acid receptor RXR-alpha-B-like	104	291	**84**	60	63	**72**	**84**	**77**
XM_038788193.1	**SPP2.3s6**	2.70	3.13		Mineralising chondrocytes	Secreted phosphoprotein 24-like	27	279	1	0	1	***518***	***341***	***182***
XM_038815678.1	BCL6	1.16		1.28		BCL6A transcription repressor a	107	269	62	98	105	***157***	***223***	114
XM_038792863.1	**scpp**	3.62	4.11	2.85	Leurs et al., 2022	Secretory calcium-binding phosphoprotein	16	252	6	195	173	***1031***	***907***	***459***
XM_038788227.1	**SPP2.3s4**	1.77	1.96		Mineralising chondrocytes	Secreted phosphoprotein 24-like	25	161	1	0	0	***260***	***367***	71
Cluster-281.0	Atg2b	1.44	1.31	1.48		Autophagy related 2b	47	153	**32**	24	24	**36**	**34**	**34**
XM_038788208.1	**SPP2.3s3**	2.12	2.42		Mineralising chondrocytes	secreted phosphoprotein 24-like	17	140	1	0	0	***458***	***575***	102
XM_038789767.1	MLXIP	1.24	1.03	1.37		MLX(Max-like protein X) interacting protein; role in transcriptional activation of glycolytic target genes	45	133	14	38	**51**	**47**	**46**	40
XM_038781460.1	**COCH**	2.92	3.00	2.31	Spinal cord	Coagulation factor C homologue, cochlin; control of cell shape and motility in the trabecular meshwork	12	130	11	27	0	***217***	***408***	***632***
Cluster-18614.1	**tgfb2**	1.36	1.90		Chondrocyte; spinal cord	Transforming growth factor beta 2	32	111	293	30	65	***1049***	***1071***	***463***
Cluster-14702.0	Smcr8a	1.15	1.29			Part of guanyl-nucleotide exchange factor complex that regulated autophagy	34	97	**34**	23	25	**30**	**32**	**29**
Cluster-2778.0	Tmem25	1.39	1.55			Transmembrane protein 25; regulation of neuronal excitability	25	84	**8**	3	**5**	**5**	**5**	**6**
XM_038783788.1	Uts2r	1.36	1.05	1.52		Urotensin-2 receptor-like	24	78	7	10	11	**15**	**33**	10
XM_038784999.1	**Uncharacterised**	3.58	3.67	3.36	Spinal cord	Uncharacterised protein	5	74	72	20	1	***217***	***227***	***124***
XM_038816355.1	Tnk2	1.16	1.04	1.21		Tyrosine kinase, non-receptor, 2b	25	69	12	13	9	**21**	**23**	**15**
Cluster-15354.0	Fzd9	1.36		1.31		Frizzled class receptor 9; receptor for Wnt signalling proteins	18	67	2	1	2	**23**	**20**	**13**
XM_038812531.1	Itih3	1.04	1.18			Inter-alpha-trypsin inhibitor heavy chain 3; carrier of hyaluronan	19	44	47	91	80	***156***	***224***	***203***
XM_038802693.1	KCTD4	2.11	1.99	2.15		Potassium channel tetramerisation domain containing 4	5	29	**8**	4	2	**6**	**6**	3
XM_038777944.1	FAM20A	1.18	1.07	1.25		Golgi associated secretory pathway pseudokinase	9	25	**14**	**17**	**17**	**16**	**14**	**13**
XM_038790369.1	lrrc3	1.35	1.15	1.13		Leucine rich repeat containing 3	4	13	3	0	2	**5**	**4**	**6**
Cluster-16311.0	skida1	1.73		1.94		SKI/DACH domain-containing protein 1; regulation of transcription	2	11	1	0	1	**4**	**4**	2
XM_038794066.1	gpr54	1.37	1.27	1.02		G protein-coupled receptor 54; receptor for KiSS1, metastasis suppressor protein	3	10	1	1	4	**11**	**5**	**7**
XM_038778800.1	PTGIR	2.52	2.58	2.17		Prostaglandin D2 receptor-like	1	8	1	**10**	**7**	**6**	**5**	**7**
XM_038789311.1	Cycs	1.38		1.25		Cytochrome C, somatic	2	7	3	2	3	**98**	**154**	39
XM_038820567.1	SPAM1	2.01	2.50			Sperm adhesion molecule 1	1	4	3	4	4	**21**	**30**	**24**
XM_038776212.1	Yrdc	2.98	3.65			Threonylcarbamoyl-AMP synthase	0	1	0	**17**	1	**37**	**47**	6

**Fig. 3:**
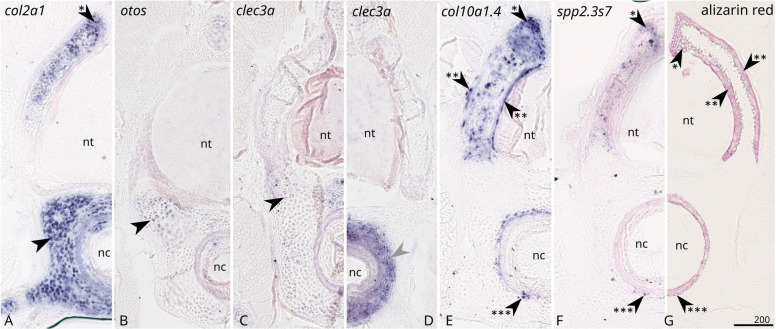
Expression of selected genes (**A**–**F**, names indicated on the top of each panel) by in situ hybridisation on transversal sections of 8 cm embryo vertebra of the small-spotted catshark *Scyliorhinus canicula*. **G** Red alizarin staining showing calcium deposition in the vertebral tissues: extracellular matrix of chondrocytes in the neural arches (black arrowhead with one asterisk, also see expression in **A**, **E**, **F**), in the fibrous perichondrium of the neural arches (black arrowhead with two asterisks, also see expression in **E**), and in a fibrous and cartilaginous sheath in the vertebral body (black arrowhead with three asterisks, also see expression in **D**, **F**). Expression in non-mineralising chondrocytes of the vertebral body indicated with a black arrowhead (**A**–**C**), expression in fibrous intervertebral area is shown with a grey arrowhead (**D**). nt: neural tube; nc: notochord. Scale bar: 200 µm

**Fig. 4:**
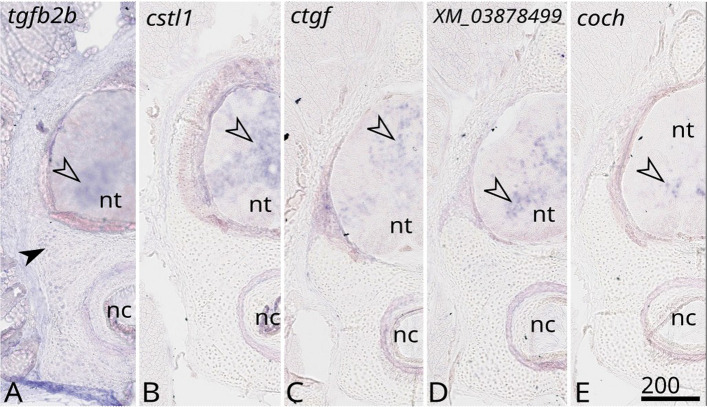
Expression of selected genes (names indicated on the top of each panel) by in situ hybridisation on transversal sections of 8 cm embryo vertebra of the small-spotted catshark *Scyliorhinus canicula* with expression in the neural tube (open arrowhead) and expression in non-mineralising chondrocytes of vertebral body (black arrowhead). nt: neural tube; nc: notochord. Scale bar: 200 µm

In situ hybridisation on cross sections of mineralising anterior vertebrae showed that three genes annotated as *clec3a* (XM_038806248.1), *otos* (XM_038816254.1), and the cluster-18614.1 were expressed in all chondrocytes in the cartilaginous tissue, comparable to the expression of *col2a1* (Figs. 3 and 4).

Phylogenetic reconstructions confirmed the 1-to-1 orthology of XP_038662176.1 (protein sequence predicted from XM_038806248.1) with the mammalian *Clec3a* coding gene (Additional file 4: Fig. S1), suggesting conserved function of Clec3a in mature chondrocyte homeostasis [32]. Phylogenetic reconstruction identified the cluster-18614.1 as *tgfb2B* coding gene (Additional file 5: Fig. S2), one of two Tgfb2-coding genes in gnathostomes previously identified and named *Tgfb2A* and *Tgfb2B* [33]. This also supports ancient conservation of both Tgfb2 proteins in chondrocyte homeostasis [34]. Phylogenetic reconstruction including XP_038672192.1 (protein sequence predicted from XM_038816254.1) confirmed its annotation as the 1–1 ortholog to the mammalian Otospiralin coding gene (Additional file 6: Fig. S3), despite the existence of a second gnathostome paralog. The observed expression of *otos* in chondrocytes was unexpected as the mammalian Otospiralin protein has restricted function in the ligament of the inner ear [35]. One of the zebrafish otospiralin genes was found expressed in the notochordal cells further suggesting a potential ancestral function in conjunctive/skeletal tissue development [36]. Finally, the other tested genes were found expressed in the neural tube (the uncharacterised *XM_038784999.1*, *coch*,* ctgf*, and *cstl1*; Fig. 4), showing that our dataset also included conserved central nervous system genes (*ctgf* in [37]; *cstl1* in [38]; *coch* in [39]) (Fig. 4). None of the tested genes showed a specific expression in mineralising chondrocytes except in the case of the Spp2-related sequences (Fig. 3 and see below).

In the ‘col2a1 normalised’ differential expression analysis, we also extracted 178 genes that showed down-regulation over the time of vertebral mineralisation. Among them, 74 had an annotated function in the development of the extracellular matrix (Additional file 7: Table S10), including major structural proteins produced by chondrocytes or perichondral cells: fibrillar (*col1a1*; *col1a2*; *col5a1*; *col5a2*; *col11a1*; *col27a*) and non-fibrillar collagens (*col6a1*; *col6a2*; c*ol6a3*; *col9a1*; c*ol9a2*; *col12a1*; *col16a1*) and collagen synthesis and assembly proteins (annotated as *loxl3b*; *plod1*; *p3h1*; *p4ha3*; *fkbp7*, *10* and *14*; *znf469*; *tfpi*; *colgalt2*; *lama4* and three Serpin genes). One of the cartilaginous fish Type X collagen coding gene (XM_038798850.1 previously identified as *col10a1.4*) was downregulated between these time points while it has been shown to be expressed by early mineralising chondrocytes [17]. This observation suggests expression of this gene comes upstream of chondrocyte differentiation into mineralising chondrocyte and is then downregulated in differentiated mineralised chondrocytes while still being strongly expressed in 8 cm vertebrae (Fig. 3E). The apparent downregulation of *col10a1.4* expression might also result from the higher proportion of proliferating chondrocytes in 8 cm vertebrae. Also in the downregulated genes were several proteoglycans, notably Agrecan (*acan*), many genes of the Small Leucin Rich Proteoglycan (SLRP) family (*aspn*, *ecm2*, *dcn*, *ecm2*, *fmod*, *prelp*, *lum*; see [21] for nomenclature) and several genes annotated as coding for proteins involved in proteoglycan synthesis and assembly (*gpc5*; *gfpt2*; *xylt1*; *chst11*). Expression of these genes might characterise the transcriptomic signature of early chondrocytes and perichondral cells at a stage preceding the mineralisation of their extracellular matrix, especially several transcription factors (*nkx3.2*, *runx2*, *mdfi* and *trps1*) and other proteins known to mediate chondrocyte differentiation (*ror2* and *igsf10*) or even osteoblast differentiation (*fndc3b*).

### A novel gene family is expanded in cartilaginous fishes and involved in cartilage mineralisation

**Fig. 5:**
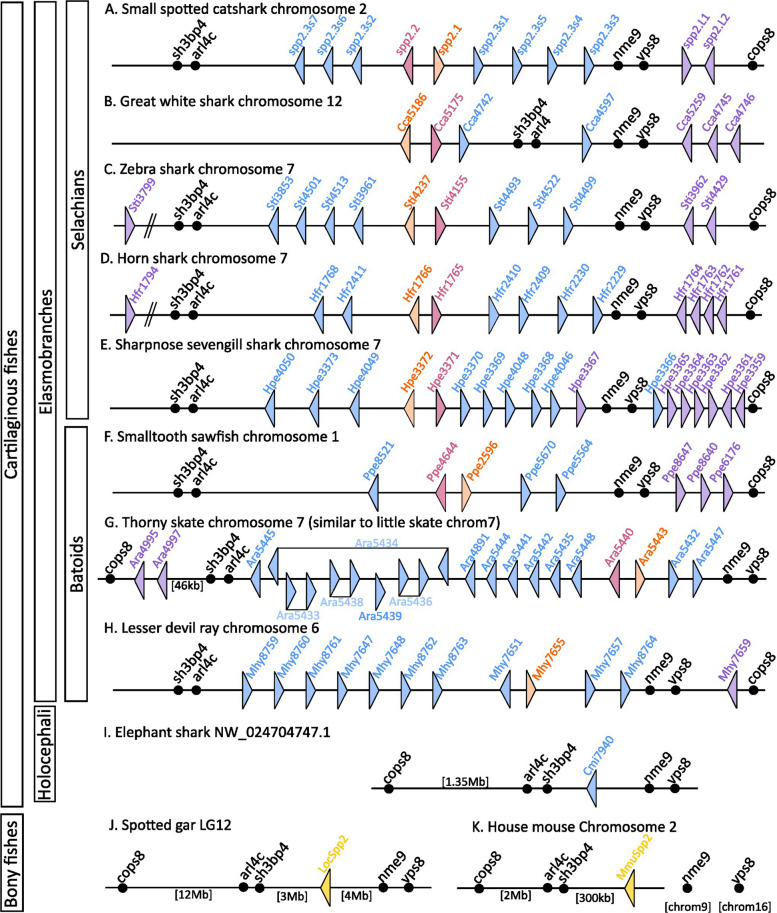
Conserved synteny between selacians (**A**–**E**) and batoids (**F**–**H**) with osteichthyans (**J**–**K**) and the elephant shark (**I**). The colour code is defined by clades identified in the phylogeny (see Fig. 6). Some predicted transcripts are interpreted as an association of two successive spp2 coding sequences and are represented as two triangles linked by a line (e.g. Ara5433)

A striking feature of the differential expression results was the identification of seven different genes, all annotated as *spp2* homologs (Secreted Phosphoprotein 2) and all strongly to mildly upregulated along the mineralisation process and expressed in chondrocytes enclosed in a mineralised matrix (Fig. 3 and Table 1). We first identified that all seven genes were located on a single genomic locus on the catshark chromosome 2, and that this locus included more than seven coding sequences belonging to this gene family. BLAST requests against the catshark genome retrieved a total of eleven sequences all located in the vicinity of the loci annotated as *nme9* and *vps8* on chromosome 2 (Additional file 8: Fig. S4). A twelfth spp2-related gene was found located on chromosome 9 (named *spp2.L3*). These catshark sequences were then used to perform BLAST requests against bony vertebrate genomes where only one single similar sequence was identified as *spp2,* also found in synteny with *nme9* and *vps8* in the spotted gar but, in mouse, only with genes located on the other side of the catshark *spp2* gene cluster (*sh3bp4* and *arl4c* genes, see Fig. 5). BLAST requests on other cartilaginous fish genomes retrieved a variable number of putative *spp2* duplicates that also were assigned to a *sh3bp4*/*arl4c*/*nme9*/*vps8*/*cops8* locus despite some apparent chromosomal rearrangement in the great white shark locus. In contrast, only one copy is present in elephant shark (Fig. 5).

**Fig. 6:**
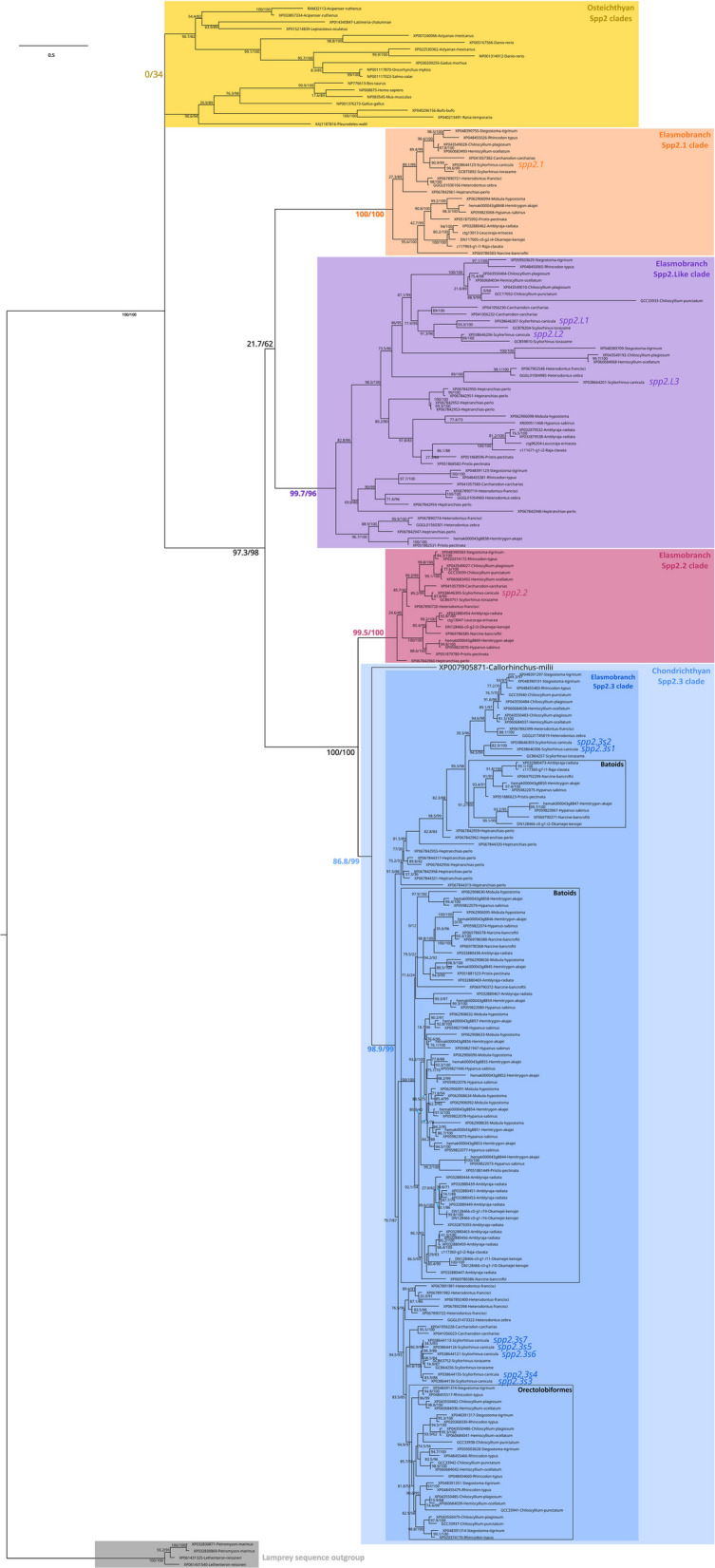
Maximum likelihood phylogenetic tree based on spp2 amino-acid sequences (230 sequences, 193 positions) with Q.mammal+R6 evolution model in IQ-TREE, and rooted with sequences from two lamprey species. Node support was evaluated with 1000 ultra-fast bootstrap replicates and SH-aLRT (UFbootstrap/SH-aLRT). Colour code: spp2.1 (orange), spp2.2 (pink), spp2.3 (blue) and spp2.Like (purple) chondrichthyan clades and spp2 (yellow) osteichthyan clade. Sequence names highlighted on the phylogeny in bigger letters locate the small-spotted catshark sequences

Sister to the osteichthyan group of sequences, the spp2 phylogeny distinguished four main clades herein called *spp2.1*, *spp2.2*, *spp2.3* and *spp2.Like*, all made of chondrichthyan sequences (Fig. 6). The clades *spp2.2* and *spp2.3* were robust sister groups, but no relationship between them and the clades *spp2.1* or *spp2.Like* was well supported. The *spp2.3* clade included the only one *spp2* copy identified in the elephant shark *Callorhinchus milii*, suggesting that the duplication events that generated the four clades predated the last common ancestor of extant chondrichthyans and that three secondary losses occurred in the elephant shark lineage. The ancestral *spp2.3* gene has undergone several additional tandem duplications at different rates in all examined elasmobranch species. The grouping of sequences found in the same species in the *spp2.3* clade indicates recent rapid duplications that occurred independently in all lineages of elasmobranch fish, leading to high sequence similarity. The seven *spp2.3* duplicates for *Scyliorhinus canicula* were named *spp2.3s1-7*: they were all upregulated in mineralised vertebrae in the DEseq analysis, although *spp2.3s3* was significantly upregulated only in posterior vertebrae (Table 1).

**Fig. 7:**
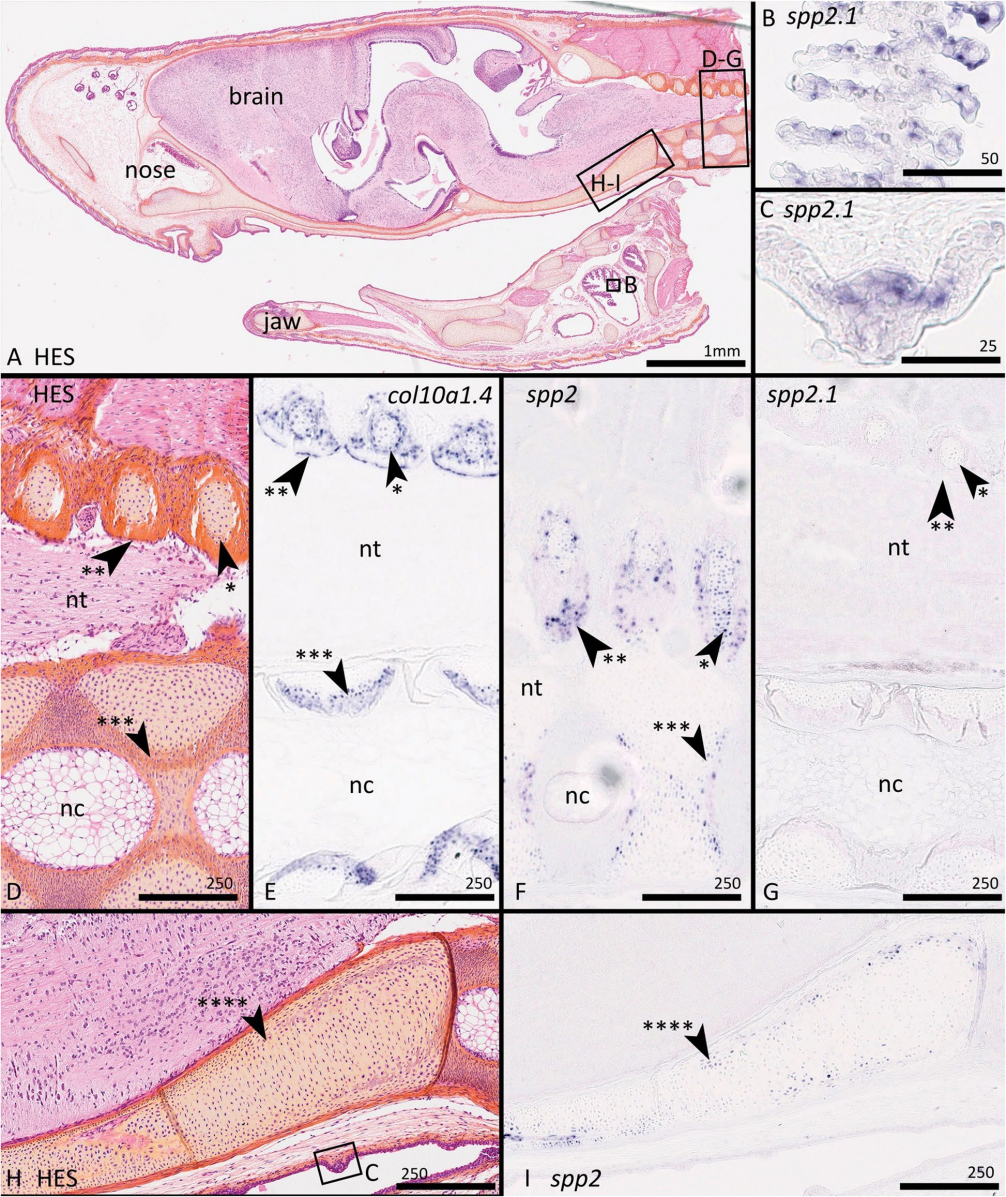
Histological staining and in situ hybridisation on successive paraffine longitudinal parasagittal sections of 8 cm long *Scyliorhinus canicula* embryo head; anterior to the left, dorsal to the top. **A**, **D**, **H** Haematoxylin–eosin-safran (HES) staining with location of following insets, namely **B** is showing gills, **C** is showing a taste bud, **D**–**G** are showing vertebrae, **H**–**I** are showing a ventral-posterior part of the chondrocranium. **B**, **C**, **G** In situ hybridisation of the *spp2.1* probe with positive expression in the epithelium of the gills (**B**) and of the taste bud (**C**) but without detected expression in vertebrae (**G**); **E** in situ hybridisation of the *col10a1.4* probe with positive expression in the perichondral mineralising cells (arrowhead with one asterisk) and in mineralising chondrocytes of the neural arches (arrowhead with two asterisks) and in cells surrounding the fibrous sheath of the vertebral body (arrowhead with three asterisks). **F**, **I** in situ hybridisation of the *spp2.3* probe with positive expression in cells similar to the *col10a1.4* expression pattern (**F**) but also in mineralising chondrocytes of the chondrocranium (arrowhead with four asterisks). nc: notochord; nt: neural tube. Scale bars in µm unless stated otherwise

**Fig. 8:**
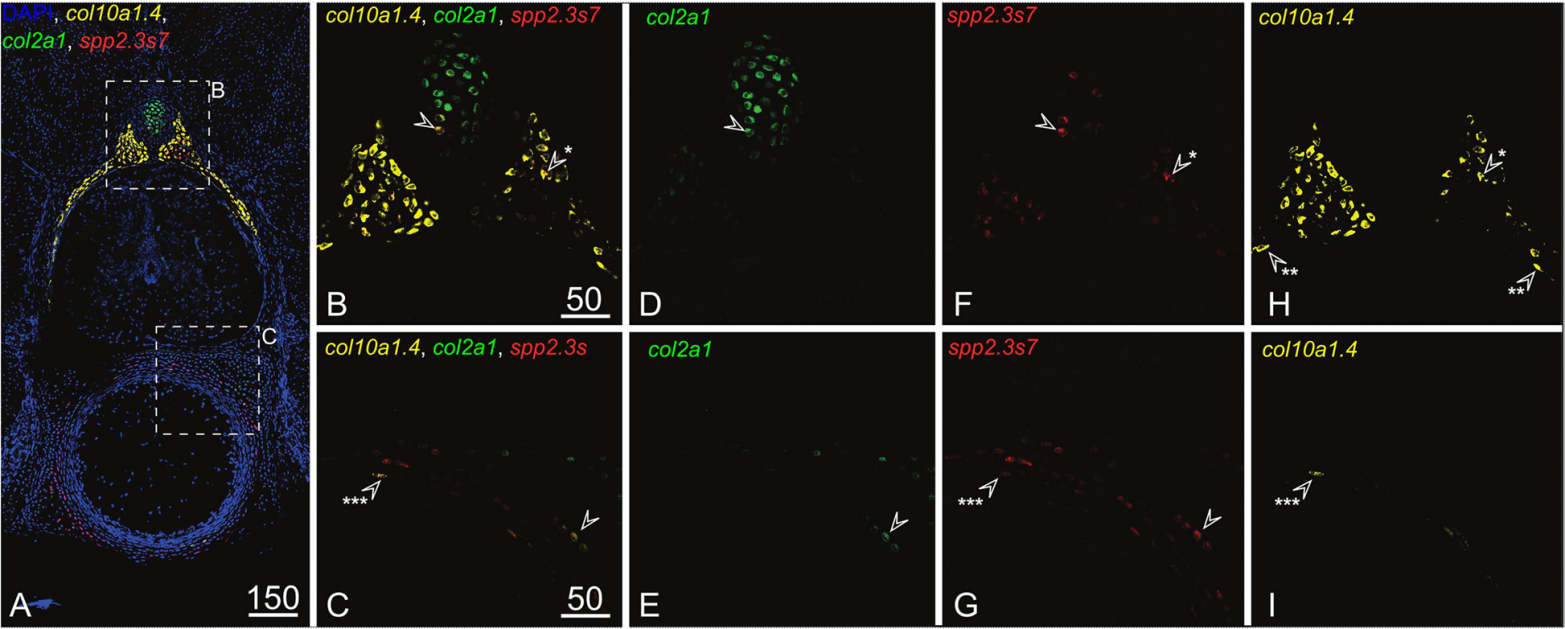
HCR-FISH showing DAPI and co-localisation of *col2a1*, *spp2.3s7* and *col10a1.4* RNAs on transverse sections of vertebrae in 8.3 cm long *Scyliorhinus canicula* embryos. **A** Maximum projection of all planes through the 14 µm cryostat section and location of following close-ups; **B**, **D**, **F**, **H** merged (**B**) or single (**D**, **F**, **H**) imaging in each canal of a single plane showing co-expression in the dorsal part of the neural arches; **C**, **E**, **G**, **I** merged (**C**) or single (**E**, **G**, **I**) imaging in each canal of a single plane showing co-expression in the dorsal lateral part of the vertebral body. Positive cells located: in the mineralising perichondrium of the neural arches are pointed by an arrowhead with one asterisk; in the mineralising cartilage of the neural arches are pointed by an arrowhead with two asterisks; in the surrounding of the mineralising fibrous sheath of the vertebral body are pointed by an arrowhead with three asterisks

Due to the sequence similarity of *spp2.3* and *spp2.2* transcripts, our RNA probe for in situ hybridisation could not be strictly gene-specific (Additional files 9: Tables S11–S12 and 10: Alignment S1). We first considered the general *spp2* probe to be able to bind most of the *spp2.3* genes, and to a lesser extent, cross-hybridise with *spp2.2*. In situ RNA hybridisation with this probe showed that at least a subset of these genes is expressed in mineralising chondrocytes of the neural arches and vertebral body of the catshark embryo (Fig. 7). This expression pattern was comparable to that obtained with the more specific probe *spp2.3s7* (> 90% similarity for *spp2.3s2* and *s6*, > 80% similarity with *spp2.3s3*/*4*/*5*, Figs. 3 and 8). Specific expression for *spp2.1* was found in the gill epithelium but not in the skeletal tissues (Fig. 7). These expression patterns were coherent with RNAseq data in adult tissues, with high TPMs in vertebrae/Meckel’s cartilage/chondrocranium for all *spp2.2* and *spp2.3* genes, and gills for *spp2.1* (Table 2). However, the low expression of two *spp2.Like* did not allow us to detect any positive staining in teeth and scales in in situ hybridisations (Table 2). A probe designed to be specific for the *spp2.2* sequence generated no detectable signal in transverse section in vertebrae of 8 cm embryos.

**Table 2:** Gene expression profiles of *spp2* gene family members in the adult small-spotted catshark. Selection of transcriptomic data (TPM values) to characterise the expression profiles of *spp2* genes in adult skeletal and non-skeletal tissues. Samples with *Z*-score > 1 are in bold. See the complete table with the 31 adult tissues/embryo stage TPM and *Z*-score values in Additional file 11 [27]

gene_name	gene_ID	Brain-OS	Spinal chord	Gills	Liver	Blood	Muscle	Denticles	Dental lamina	Meckels	Chondrocranium	Vertebrae
spp2.1	XM_038788195.1	**1680.69**	31.51	**6145.36**	0.07	0.00	1.82	898.00	518.19	121.71	45.84	11.85
spp2.2	XM_038790377.1	9.69	29.66	10.19	0.22	0.10	10.27	14.46	16.85	**347.29**	160.03	**570.04**
spp2.3s1	XM_038790378.1	0.99	3.03	0.03	0.00	0.02	0.34	0.36	0.47	**1109.06**	**372.68**	**1077.52**
spp2.3s2	XM_038790375.1	11.58	1.29	1.97	0.00	0.00	0.08	0.11	1.29	**2691.79**	**877.15**	**2378.12**
spp2.3s3	XM_038788208.1	0.62	0.61	0.06	0.00	0.04	0.00	0.05	0.16	**458.47**	102.26	**574.96**
spp2.3s4	XM_038788227.1	0.43	0.60	0.00	0.00	0.07	0.00	0.10	0.02	**259.98**	70.81	**366.51**
spp2.3s5	XM_038788196.1	2.06	2.75	0.19	0.00	0.00	0.01	0.11	0.34	**724.66**	251.39	**853.56**
spp2.3s6	XM_038788193.1	37.17	1.33	11.12	0.00	0.07	0.02	0.06	0.75	**517.90**	**182.49**	**341.31**
spp2.3s7	XM_038788185.1	13.92	1.35	8.69	0.17	0.00	0.00	0.30	1.30	**2707.59**	**1067.40**	**2677.98**
spp2.L1	XM_038790379.1	0.44	1.41	0.05	0.00	0.02	0.12	**39.16**	**19.13**	2.73	0.12	0.29
spp2.L2	XM_038790278.1	2.17	2.33	1.77	2.30	2.16	1.59	**77.69**	**63.09**	7.64	2.83	2.01
spp2.L3	XM_038808273.1	0.25	0.16	0.21	0.03	0.16	1.16	**342.01**	76.77	26.56	0.89	0.27

We generated another set of probes specific to the *spp2.3s7* sequence to be used in HCR™ Gold RNA-FISH experiment, to be compared with the expression of *col2a1* and *col10a1.4* in the different cell populations in 8 cm embryos (Fig. 8). The detected signal supports co-expression of this *spp2.3s7* gene in chondrocytes of mineralising tissues, both in the core of neural arches, but also in the surrounding of the fibrous sheath in the vertebral body (Fig. 8F–I). It also showed co-expression between *spp2.3s7* and *col2a1* in some chondrocytes located just external to these mineralising sites (Fig. 8D–G), suggesting an intermediate differentiation stage where *col2a1* is still expressed but not yet *col10a1.4*.

### Conserved protein domains in cartilaginous fish spp2 duplicates

**Fig. 9:**
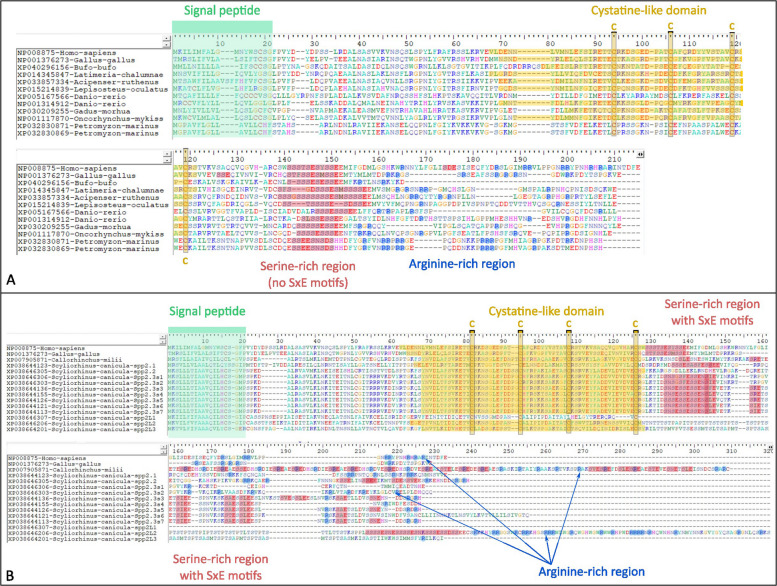
Protein sequence alignments of osteichthyan and lamprey sequences (**A**) and of human, chicken, elephant shark and catshark sequences (**B**) with highlighted identified signal peptide, cystatin-like domain (four conserved cysteines), stretches of serine defining the serine-rich region, occurrence of SxE motif, and occurrence of arginine in the C-terminal R-rich regions

The human and other bony fish spp2 protein sequences contain a signal-peptide, a cystatin-like domain with four conserved cysteines, a series of serine defining a ‘serine-rich region’ that includes the putative phosphorylation sites, and finally a C-terminal region rich in arginine that was mostly detectable in sarcopterygian and lamprey proteins (Fig. 9). The catshark sequences in the spp2.1, 2.2 and 2.3 clades also had the cystatin-like domain and were characterised by the accumulation of a large number of SxE motifs in the C-terminus of the protein in addition to the stretch of serine. The most extreme case was found in the single spp2.3 elephant shark sequence with 21 SxE motifs but devoid of the serine stretch (Fig. 9). The arginine-rich C-terminal region was obvious only in the catshark spp2.1 and the spp2.L2 sequences but also in the elephant shark sequence. spp2.Like sequences showed major divergence, with spp2.L1 missing an identifiable cystatin-like domain, and spp2.L2 having a repeated sequence (PTST/PTSTST) inserted between the cystatin-like domain and the serine-rich region.

## Discussion

### An ancestral toolkit for differentiating chondrocytes in jawed vertebrates

Our differential expression analysis retrieved a series of genes that were upregulated either after or before the mineralisation process in the vertebrae of the catshark. The transcriptomic signature of chondrocytes before this maturation step was easily comparable to known function of proteins in model species including synthesis, assembly and maintenance of collagens and proteoglycans by chondrocytes (some of them already described in the catshark [6, 17, 21]). A more precise description of this transcriptomic signature would require single-cell RNAseq and/or spatial transcriptomics data allowing to resolve cell population diversity within the vertebral zone. In contrast, the transcriptomic signature of chondrocytes in the end of the mineralisation process was much less comparable to model species as only *bgp*, *clec3a*, *otos*, *tgfb2b* and *spp2* genes could be assigned to a known function in chondrocytes or other skeletal cells in model organisms. Some known actors of the early steps of chondrocyte differentiation into mineralising chondrocytes in the catshark (eg *col10a1.4* [17] or *mgp* [18] or *sparc* [19]) were not identified in the differential expression analysis, the main reason being their expression at the initiation of mineralisation (*col10a1.4* and *mgp* [17, 18]) or the non-specificity of their expression (*sparc* is expressed strongly in younger stages in the surrounding conjunctive tissue [19]). This suggests that our sampling strategy might not have captured the initiation process, the premise of the first stage of differentiation, which would occur before the 5 cm stage. This initiation process might include the activation of *col10a1.4* by runx2 [40] prior to the 5 cm stage, explaining the downregulation of both genes between 5 and 8 cm. This observation may also result from a variation in the proportion of non-mineralising/mineralising chondrocytes between the 5 cm and the 8 cm stage: non-mineralising chondrocytes have high level of proliferation making the non-mineralised cartilage a bigger volume compared to mineralised tissue found in the surface of neural arches and of the fibrous sheath of the vertebral body (Fig. 3G). Our sampling strategy would need a better time resolution (before stage 5 cm and in between our start/end points) to generate finer descriptions of gene expression patterns in future studies. However, this initial strategy still allowed to identify some aspects of the transcriptomic signature of more differentiated cells: the *clec3a* expression upregulated in the fibrous intervertebral cells; the *spp2.3* genes expressed in the chondrocytes differentiating from their *col2a1* expression status towards a *col10a1.4* expression status, and becoming enclosed in a mineralised matrix. However, our list of 79 differentially expressed genes included 24 predicted transcripts of poor quality, and 6 non-coding RNA, that we did not investigate further for the lack of a comparative framework with model organisms. Some non-coding RNAs function in the differentiation of skeletal tissues [41–43] and a more thorough analysis of the potential homologs between the mouse and the catshark non-coding RNAs is still currently necessary to test potential conservation of these processes. The most striking result of this analysis was the robust identification of several duplicates of the *spp2* gene family in genes upregulated in the post-mineralisation state.

### Evolutionary diversification of spp2

#### Gene family expansion

Our phylogenetic reconstruction and genomic data revealed that a single *spp2* gene was maintained at a conserved locus in bony fishes, whereas several tandem duplications occurred in cartilaginous fishes in the homologous locus. At least three tandem duplications gave rise to four *spp2* clades in elasmobranchs: the *spp2.1* and *spp2.Like* clades, and the two sister *spp2.2* and *spp2.3* clades. The phylogenetic reconstruction placed the single *spp2* gene of the elephant shark within the *spp2.3* clade, indicating that all these duplications happened in early cartilaginous fishes, before the divergence between holocephalans and elasmobranchs (Fig. 6). The *spp2.1* and *spp2.2* genes were present in only one copy in each species, while *spp2.3* and *spp2.Like* occurred in several copies in some species. In the *spp2.Like* clade, duplications happened several times in very different patterns depending on the lineage (three duplicates in the catshark, up to seven in the sharpnose sevengill shark, some with a rather conserved position along the gene cluster but other duplicates were found on the other chromosomes—e.g. *spp2.L3* in the catshark). Duplications in the *spp2.3* clade were even more frequent in elasmobranch species, leading to seven copies in the catshark and up to fifteen (and probably more due to mis-annotation of tandem copies) in the skate *Amblyraja radiata* (see Fig. 5). On the contrary, a single paralog was conserved in the elephant shark with the only one *spp2.3* gene, suggesting many secondary gene losses in the lineage.

#### Functional implications

The mammalian *Spp2* encodes a secreted phosphoprotein that shares a protein domain with members of the cystatin family of thiol protease inhibitors [44]. Together with Fetuin-A and Mgp, Spp2 is a component of the calciprotein particles, a circulating Fetuin-mineral complex that buffers phosphate and calcium ions by capturing them in the bloodstream, thereby preventing them from precipitating under physiological conditions [45, 46]. The mammalian Spp2 is expressed by adult liver and periosteal cells [47] but also by embryonic liver and metanephros cells [44, 48] and has been associated with mineral metabolism through kidney function [49]. Spp2 is thought to modulate the rate and extent of bone formation [50]. In zebrafish, a large increase in spp2 protein abundance was observed during development in the skeletal extracellular matrix [51] while transgenic mice over-expressing spp2 were found to have reduced femoral and vertebral bone mineral density [52]. These contradictory results on the effect of Spp2 protein on skeletal mineralisation may be an outcome of its interaction with BMP2 (Bone Morphogenetic Protein-2) that leads to either a pro- or an anti-osteogenic effect depending on the cleavage status of the protein [53, 54]. As a consequence, understanding the function of the different proteins encoded by these duplicated genes is not a simple question in non-model organisms where protein cleavage status is not known.

The N-terminal cystatin-like domain was conserved in cartilaginous fish protein sequences, and its acidic composition is thought to be responsible for the inhibition of the basic calcium phosphate precipitation while its most C-terminal sequence is responsible for Bmp2 interaction [53, 55]. It is followed by a serine-rich (putative Fam20C phosphorylation sites [56]) and an arginine-rich C-terminal region. All cartilaginous fish *spp2* duplicates show accumulation of a large number of SxE motifs in addition to the conserved stretch of serine that follows their cystatin-like domain, and the single elephant shark spp2.3 reaches thirteen motifs while it lost the original stretch of serine (Fig. 9). Phosphorylation by Fam20C allows Fetuin A to circulate in the blood [45], and this characteristic could therefore be shared with Spp2 proteins, including cartilaginous fish duplicates. The catshark spp2 proteins do not seem to show a C-terminal arginine-rich region, except in the case of spp2.1, suggesting functional divergence between this duplicate and the spp2.2 or spp2.3 proteins. Overall, protein sequence conservation suggests shared function in the interaction with Bmp2 and an ability to interact with calcium-phosphate particles in all jawed vertebrates, and for all duplicates of spp2.1, spp2.2 and spp2.3 clades, excluding spp2.Like sequences that display strong sequence divergence and partial loss of the cystatin-like domain. The lamprey sequences display sequence divergence that does not allow the recognition of the cystatin-like domain with InterProScan, but they show several conserved features including all conserved cysteines of the cystatin-like domain, the serine-rich stretch and the arginine-rich C-terminal region. A description of the expression pattern and location of the lamprey proteins might give insight into the original function of spp2 proteins in early vertebrates before the evolution of a mineralised skeleton.

#### Evolution of expression

The Spp2 and Fetuin-A genes are known to be mostly expressed by liver cells with their protein products circulating in the blood where they interact with calcium, making both the inhibition of vasculature possible and providing calcium to the mineralising sites through the blood stream [57, 58]. As both proteins share sequence similarity (in the cystatin-like domain), previous studies have proposed that both genes emerged from the duplication of an ancestral gene [57] whose protein product would then have displayed both a capacity to bind calcium and the characteristic to be produced from liver cells and secreted in the blood stream.

In the small-spotted catshark, none of the Spp2 duplicates were expressed by liver cells, suggesting evolutionary events at their regulatory sites after duplication. The expression of *spp2.Like* genes appeared restricted to epithelial sites (scales and dental lamina) where their putative function is difficult to apprehend. The expression *spp2.1* was found highest in the brain/olfactory epithelium and in the gill samples. This latter site of expression might be functionally similar to the kidney expression of Spp2 in mammals, as ion transport regulation occurs in gills of cartilaginous fishes [49, 59, 60] or might be the result of neo-functionalisation of this duplicate. A striking aspect in the evolution of expression patterns is the transcription of all duplicates of *spp2.3* and *spp2.2* genes in skeletal units. RNAseq data of the lower jaw (Meckel’s cartilage) of the thornback ray *Raja clavata* [17] also show expression of all four identified Spp2 duplicates (see Fig. 6) with TPM values 35, 276, 49 and 119 for respectively c111671_g1_i2 (*spp2.L* duplicate), c117360_g3_i2 (*spp2.3* duplicate), c117963_g1_i1 (*spp2.1* duplicate) and c117360_g7_i1 (*spp2.3* duplicate), indicating high levels of expression for the ray *spp2.3* and *spp2.2* duplicates. As a consequence, this specialised expression pattern appears to be a derived feature, maybe shared by all cartilaginous fishes. This gene family therefore seems to have evolved into a major gene family involved in cartilage mineralisation, both through tandem duplications generating multiple copies that may be selected for the production of higher quantities of the functional protein [61], and through the evolution of regulatory sequences leading to a capacity of chondrocytes to directly express all members of the gene family, instead of fixating these proteins from the blood stream. Another hypothesis that should be tested further is the potential correlation between the degree of mineralisation of each species with the number of spp2 copies. A rather simplistic observation is the presence of a single copy in *C. milii,* a species with relatively low levels of mineralisation [62], to eighteen copies in *A. radiata* whose skeleton is extremely mineralised [63]. The loss of all but one *spp2.3* gene copies in the elephant shark might result in its poorly mineralised skeleton. However, multiplications of the putative SxE phosphorylation site might also have evolved to inhibit mineralisation (as was shown for hyperphosphorylation of Spp1 in mammals [64, 65]). The evolutionary scenario linking Spp2 proteins to the evolution of mineralised tissues now needs more protein-level functional insights in both the cartilaginous and bony fishes.

## Conclusions

Our results shed light on contrasting aspects of the evolution of skeletal tissues in vertebrates. Our transcriptomic data highlighted some highly conserved features in the development and differentiation of chondrocytes in cartilaginous and bony fishes, dating back from their last common ancestor, more than 400 million years ago. However, these transcriptomic data also identified a derived gene family where multiple tandem duplications and putative regulatory sequence evolution have generated a series of chondrocyte-specific genes involved in the mineralisation of their extracellular matrix. This situation is quite comparable to the evolution of the secretory calcium-binding phosphoprotein (SCPP) genes [66] that duplicated in bony fishes as multiple genes specialised in calcium interaction, both associated with the mineralisation of different extracellular matrices (bone, dentine, enamel) and to other fluid compartments (saliva, tears and milk in mammals) [67]. Bone loss in cartilaginous fishes therefore involved gene loss (e.g. Osterix (Sp7) that is a key transcription factor for osteoblast differentiation [68]) but also divergence of bone-related genes that have been recruited in the evolution of their mineralised cartilage—e.g. the Spp2 gene family.

## Methods

### Embryo collection

The small-spotted catshark *Scyliorhinus canicula* embryos were collected from laid eggs by a Mediterranean population of adult females housed at the Observatoire Océanologique of Banyuls-sur-mer, France. Embryos were raised in small tanks with seawater at 16–18 °C and euthanised by overdose of tricaine (MS222, Sigma) at appropriate stages, between late stage 31 (total length of 5 cm) and stage 33 (total length of 8 cm) [28, 69].

### Bulk RNAseq on vertebrae before and after the initiation of mineralisation

Vertebrae of *Scyliorhinus canicula* male embryos were dissected at two developmental time points, pre- (4.8, 4.9, 5.3 cm total length) and post- (8.0, 8.3, 8.5 cm total length) mineralisation of the vertebrae [28]. Each individual was sampled from an anterior (abdominal, level of pectoral fins) and posterior (caudal, just posterior to the pelvic fins) part of their spine (Fig. 1). Total RNA was isolated with ReliaPrep RNA tissue Miniprep system according to the supplier’s instructions (Promega), their quality was verified using Bioanalyzer 2100 (Agilent Technologies) and RIN levels were all above 7.6. Twelve RNAseq libraries were prepared using TruSeq mRNA library preparation kit (Illumina) with 1 μg scale total RNA per library. Single-end (100 bp) sequencing of RNA libraries was performed using Illumina NovaSeq 6000 by the Montpellier MGX sequencing platform (http://www.mgx.cnrs.fr/). The depth of sequencing was between 32.6 to 46.4 million reads, after Illumina filters (Table 3).

**Table 3:** Number of reads obtained for each library. PF: Number of clusters after Illumina filters (‘Purity Filter’). *VA*, vertebrae, anterior; *VP*, vertebrae, posterior; 4–8: total length of the embryo 4.8 cm

Library	PF
VA_4-8	32,634,963
VA_4-9	39,897,012
VA_5-3	32,626,755
VA_8-3	40,116,755
VA_8-5	32,643,547
VA_8	34,482,856
VP_4-8	41,565,409
VP_4-9	43,717,126
VP_5-3	35,579,708
VP_8-3	36,493,137
VP_8-5	46,416,755
VP_8	38,798,824
**Total**	**454**,**972**,**847**

Single-end RNAseq reads (NCBI Bioproject accession number PRJNA1337120) were cleaned with fastp [70] and then mapped onto the reference gene model [31] using Kallisto [71]. Count values and TPM values for each gene and each library were recovered (Additional file 1: Tables S1,S3).

To control for different proportions of cartilage between samples, the count value for each gene was normalised by the count value of a known specific chondrocyte marker, the collagen2a1 gene (*col2a1*; XM_038786372.1; [6, 72]) in each sequenced library so that the ‘col2a1-normalised count’ = gene count*(1473/TPMcol2a1), where 1473 is the median TPM value for *col2a1* in all libraries, and TPMcol2a1 is the TPM value for *col2a1* in the library being normalised (Additional file 1: Tables S2,S4). We considered the *col2a1* gene as the best proxy for the number of chondrocytes at each stage of development despite our observation of lower level of expression in mineralising chondrocytes compared to non-mineralising chondrocytes (Fig. 8), similar to what is known in mammals [73]. We chose *col2a1* as the best available proxy even though it probably underestimates the total quantity of chondrocytes in latter stages.

The expression data were imported into R using the tximport package [74]. The differential expression analysis was performed using the DESeq2 R package [75], by placing young and old embryos in opposition. Differentially Expressed Genes (DEGs) were identified using standard DESeq2 parameters using lfcShrink() function with the ‘apeglm’ algorithm [76]. We performed the DESeq2 analysis on (see Fig. 2):

[analysis 1]: Set of count values data before the col2a1 normalisation.

[analysis 2]: Set of count values data after the col2a1 normalisation.

[analysis 3]: Set of count values data considering only anterior vertebrae without col2a1 normalisation.

[analysis 4]: Set of count values data considering only anterior vertebrae with col2a1 normalisation.

[analysis 5]: Set of count values data considering only posterior vertebrae without col2a1 normalisation.

[analysis 6]: Set of count values data considering only posterior vertebrae with col2a1 normalisation.

### Phylogenetic reconstructions

‘*spp2*’ annotated coding sequences from *Scyliorhinus canicula* identified in the DEseq analysis were used as queries for BLASTP and TBLASTN conducted on each of the target species. The nucleotide and protein sequences of *spp2* were extracted from NCBI (*Scyliorhinus canicula* [sScyCan1.1, GCA_902713615.1], *Carcharodon carcharias* [sCarCar2.pri, GCA_017639515.1], *Rhincodon typus* [sRhiTyp1.1, GCA_021869965.1], *Heterodontus francisci* [sHetFra1.hap1, GCA_036365525.1], *Heptranchias perlo* [sHepPer1.hap1, GCA_035084215.1], *Stegostoma fasciatum* [sSteFas1.1, GCA_022316705.1], *Chiloscyllium plagiosum* [ASM401019v2, GCA_004010195.1], *Amblyraja radiata* [sAmbRad1.1.pri, GCA_010909765.2], *Mobula hypostoma* [sMobHyp1.1, GCA_963921235.1], *Pristis pectinata* [sPriPec2.1.pri, GCA_009764475.2], *Callorhinchus milii* [IMCB_Cmil_1.0, GCA_018977255.1], *Latimeria chalumnae* [LatCha1, GCA_000225785.1], *Lepisosteus oculatus* [LepOcu1, GCA_000242695.1], *Acipenser ruthenus* [ASM1064508v1, GCA_010645085.1]*, Gallus gallus* [bGalGal1.mat.broiler.GRCg7b, GCA_016699485.1], *Bos taurus* [ARS-UCD1.2, GCA_002263795.2], *Mus musculus* [GRCm39, GCA_000001635.9], *Homo sapiens* [GRCh38.p14, GCA_000001405.29], *Petromyzon marinus* [kPetMar1.pri, GCA_010993605.1]), from transcriptomes available on Squalomix (github.com/Squalomix/sequences; *Chiloscyllium punctatum*, *Hemitrygon akajei*, *Okamejei kenojei*, *Scyliorhinus torazame*), from a transcriptome available on Skatebase (skatebase.org; *Leucoraja erinacea*, LS-transcriptB2), and from a locally assembled transcriptome (*Raja clavata*, [17]). All sequences used in this study are detailed with IDs and origin in the Additional file 12: Tables S14-S17. Protein sequences were aligned with MAFFT (v.7.511, [77]; standard parameters and –auto strategy, Additional file 13: Alignment S2), alignments were then cleaned by discarding the positions with more than 50% gaps. Our final alignment used for subsequent phylogenetic reconstruction was 193 amino acids long for 230 sequences and is available in the Additional file 14: Alignment S3. Phylogenetic analyses were performed to infer the evolutionary history of these genes and were reconstructed in Maximum Likelihood using IQ-Tree (v1.6.12; [78]) using the best model of amino-acid evolution ModelFinder [79] and the Bayesian Information Criterion [80]. Node support was estimated by performing a thousand ultra-fast bootstrap replicates (UFBoot; [81]), and single branch tests (SH-aLRT; [82]). The same procedure was used to reconstruct the phylogenies of the Otos, Clec3 and Tgfb genes (Additional file 12: Tables S14-S17 and Additional files 15–17: Alignements S4-S6).

### Synteny analyses

The synteny of *spp2* paralogs was reconstructed for eleven species whose genomes were explored through the genome data viewer tool in NCBI: the domestic mouse *Mus musculus* (GRCm39), the spotted gar *Lepisosteus oculatus* (LepOcu1), elephant shark *Callorhinchus milii* (IMCB_Cmil_1.0), small-spotted catshark *Scyliorhinus canicula* (sScyCan1.1), great white shark *Carcharodon carcharias* (sCarCar2.pri), zebra shark *Stegostoma tigrinum* (sSteTig4.hap1), horn shark *Heterodontus francisci* (sHetFra1.hap1), the sharpnose sevengill shark *Heptranchias perlo* (sHepPer1.hap1), smalltooth sawfish *Pristis pectinata* (sPriPec2.1.pri), thorny skate *Amblyraja radiata* (sAmbRad1.1.pri) and lesser devil ray *Mobula hypostoma* (sMobHyp1.1).

### *mRNA *in situ* hybridisation*

In situ hybridisations were performed on 14 μm thick cryostat sections of 8 cm long *Scyliorhinus canicula* embryos, cut transversely in the body trunk, at the level of the pectoral fins or longitudinally in the head, or on paraffine sections of 10 µm thickness cut in the longitudinal parasagittal plane of an 8 cm long *Scyliorhinus canicula* embryo. Small-spotted catshark *col2a1* [6], *col10a1.4* [17], *coch*,* clec3a*,* ctgf*,* cstl1*,* otos*,* tgfb2*,* uncharacterised XM_038784999.1* and *spp2* sequences were used to design primers (Additional file 18: Table S18). For the *spp2* gene family, given the level of similarity in the nucleotide sequences, a first probe (spp2 probe) was designed to recognise all the *spp2.3* duplicate transcripts (between 95 and 99% sequence similarity), and maybe also the *spp2.2* transcript (~ 83% sequence similarity) (see Additional file 9: Tables S11–S12 and Additional file 10: Alignement S1). This probe includes a 5′ extremity that starts beyond the predicted transcript sequences as it was first designed from a locally assembled transcriptome (Additional file 10: Alignement S1).

In a second step, *spp2.1*, *spp2.3s2*,* spp2.3s7* and both *spp2-like* sequences with flanking T7 (3′ end) and SP3 (5′ end) sites were ordered for DNA matrix synthesis by the TWIST Bioscience company (https://www.twistbioscience.com/) (Additional file 18: Table S18). The *spp2.3s2* and *spp2.3s7* probes were chosen in a location of lower similarity with *spp2.2* so that they are more specific to the *spp2.3* transcripts (Additional file 9: Tables S11–S12 and Additional file 19: Alignment S7). Each PCR product and synthesised matrix were then used as templates for the synthesis of antisense DIG riboprobes [18]. All subsequent in situ hybridisation steps on cryostat sections were performed as previously described [18], and those on paraffin sections were as previously described in [83]. Slides were scanned on a Hamamatsu NanoZoomer (Montpellier RIO Imaging facility, INM Optique) with a 40 × objective.

For Hybridisation-chain reaction (HCR) fluorescent in situ hybridisation (FISH), a probe set targeting the coding sequence of each gene was custom-designed by Molecular Instruments. For each gene, the accession number, probe set size and amplifier/AlexaFluor are summarised in Additional file 20: Table S19. The HCR experimental procedure on cryostat sections follows the manufacturer’s instructions, with an added pre-fixation step for 10 min in 4% PFA. Slides were imaged using a Leica Stellaris 8 confocal (Montpellier RIO Imaging facility, DIADE) with a PL APO 40 ×/1.10 W CORR CS2—Long Distance objective.

### Protein domain identification

Protein domains were identified through the InterPro interface for InterProScan (https://www.ebi.ac.uk/interpro/search/sequence/). SxE putative phosphorylation sites were identified visually.

## Supplementary Information

Additional file 1. Tables S1-S4. Output of reads mapping onto the reference gene model [31] using Kallisto [71] in each of the 12 libraries. Each library is denominated following: VA for anterior vertebra, VP for posterior_ x-x size of the embryo_Sx: reference number of the library_valueType. Table S1 – count values; Table S2 – count values after Col2a1normalisation; Table S3 –TPM values; Table S4 – TPM values after Col2a1 normalisation.

Additional file 2. Tables S5-S8. Output of overrepresentation tests for GO terms with comparisons with the full set of annotated human proteins. Table S5 – GO terms of 3076 genes differentially expressed in the original count dataset; Table S6 – GO terms of 2998 genes differentially expressed in the col2a1-normalised count dataset; Table S7 – List of 654 genes defined as ‘skeletal markers’ (see Main text); Table S8 – GO terms of 654 skeletal marker genes.

Additional file 3. Table S9. Statistically differentially expressed genes (8cm versus 5cm long embryo). List of statistically differentially expressed genes from the DEseq analyses given as Log2(FoldChange) values in each comparison setup (see Additional File 2: Table S7): 'all_raw' is comparing raw count values and pooling anterior vertebra (VA) and posterior vertebra (VP) for both developmental stages ; 'all_normalised' is comparing col2a1-normalised count values and pooling anterior vertebra (VA) and posterior vertebra (VP) for both developmental stages ; 'VA_raw' is comparing raw count values and comparing only anterior vertebra (VA) for both developmental stages; 'VA_normalised' is comparing col2a1-normalised count values and comparing only anterior vertebra (VA) for both developmental stages;'VP_raw' is comparing raw count values and comparing only posterior vertebra (VP) for both developmental stages; 'VP_normalised' is comparing col2a1-normalised count values and comparing only anterior vertebra (VP) for both developmental stages. The mean col2a1 normalised TPM values are given for each for 5cm long embryos and 8 cm long embryos.

Additional file 4. Figure S1. Phylogenetic relationships of Clec3a-related sequences in vertebrates obtained by maximum likelihood (best fit model according to BIC: Q.plant+I+G4; 18 sequences, 230 amino-acid positions) rooted by the lamprey *Lethenteron reissneri* closest sequences. Highlighted clades represent gnathostome orthology groups, SH-aLRT/UFBoot values for internal nodes are shown on each node. The small spotted catshark sequence to be identified is XP_038652511.1 in bold and belongs to the gnathostome Clec3a group of orthology.

Additional file 5. Figure S2. Phylogenetic relationships of Tgf-beta sequences in vertebrates obtained by maximum likelihood (best fit model according to BIC: JTT+I+G4; 77 sequences, 502 amino-acid positions) rooted by the Tgf-beta1 vertebrate clade as in [33]. Highlighted clades represent gnathostome orthology groups, SH-aLRT/UFBoot values for internal nodes are shown on each node. The small spotted catshark sequence to be identified is Cluster-18614 in bold and belongs to the gnathostome TGF-beta2B group of orthology (gene nomenclature follows [33]).

Additional file 6. Figure S3. Phylogenetic relationships of Otos sequences in vertebrates obtained by maximum likelihood (best fit model according to BIC: JTT+G4; 14 sequences, 131 amino-acid positions) rooted by the lamprey *Lethenteron reissneri* closest sequence. Highlighted clades represent gnathostome orthology groups, SH-aLRT/UFBoot values for internal nodes are shown on each node. The small spotted catshark sequence to be identified is in bold and belongs to the gnathostome Otospiralin group of orthology.

Additional file 7. Table S10. Significantly differentially expressed genes in 8cm *versus* 5cm long embryo vertebrae (log2FC < -1 in different analyses see Figure 2). TPM 5 cm: mean value of col2a1 normalised anterior and posterior vertebra in 5 cm embryo. Genes in bold are published.

Additional file 8. Figure S4. *Scyliorhinus canicula *spp2 gene cluster on chromosome 2. The colour code follows the one of clades in the phylogeny (see Figure 6).

Additional file 9. Tables S11-S12. Table S11 – Similarity matrix obtained from the aligned sequences of *spp2.3* duplicates (*spp2.3s1* to *s7*), and the *spp2.2* sequence with the ‘spp2’. Table S12 – Similarity matrix obtained from the aligned sequences of *spp2.3* duplicates (*spp2.3s1* to *s7*), and the ‘spp2.3s2’ and ‘spp2.3s7’ sequences of the matrix for RNA probe synthesis.

Additional file 10. Alignment S1. The transcript sequences of *spp2.3* duplicates,* spp2.2* gene and of the spp2 probe matrix used for ISH were aligned with ClustalW. As the ‘spp2’ matrix extends upstream of the predicted start of transcription, 5’genomic sequences (NCBI reference genome sScyCan1.1) were added to the XM_ predicted sequences to generate the full alignment. The 3’ end of the alignment is defined at the end of the probe matrix sequence.

Additional file 11. Table S13. Transcriptomic data (TPM values) to characterise the expression profiles of *spp2* gene family in different embryo stages and adult tissues in the small-spotted catshark. Samples with Z-score > 1 are in bold (from [27]).

Additional file 12. Tables S14-S17. All sequences used in this study detailed with IDs, origin and coordinates in their reference genome. Table S14 – Spp2 sequences, the spp2 colour code comes from the phylogeny study (see figure 6). Table S15 – Otos sequences; Table S16 – Clec3 sequences; Table S17 – Tgfb sequences.

Additional file 13. Alignment S2. Alignment of Spp2 sequences with MAAFT.

Additional file 14. Alignment S3. Alignment of Spp2 sequences with MAAFT, cleaned for all positions with less than 50% sites on a position, and used for the phylogenetic reconstruction.

Additional file 15. Alignment S4. Alignment of Otos sequences by CLUSTALW.

Additional file 16. Alignment S5. Alignment of Clec3 sequences by CLUSTALW.

Additional file 17. Alignment S6. Alignment of Tgfb sequences by CLUSTALW.

Additional file 18. Table S18. forward and reverse primers and matrices used for *in situ* hybridisation experiments.

Additional file 19. Alignment S7. the transcript sequences of *spp2.3* duplicates,* spp2.2* gene and of the spp2.3s2 and spp2.3s7 probe matrices used for ISH were aligned with ClustalW. The 5’ and 3’ ends of the alignment are defined by the extremities of the probe matrix sequences.

Additional file 20. Table S19. HCR probes.

## Data Availability

Sequence data that support the findings of this study have been deposited in the European Nucleotide Archive with the primary accession code PRJNA1337120.

## Abbreviations

DEseq: Differential expression analysis
HCR FISH: Hybridisation-chain reaction, fluorescent in situ hybridisation
GO terms: Gene Ontology terms
RNAseq: RNA sequencing
Spp2: Secreted phosphoprotein 2
TPM: Transcripts per million
