# Supplementary figures and images for: Transcriptomic signature of differentiating catshark cartilage unravels the co-evolution of the Spp2 gene family with skeletal mineralisation in cartilaginous fish
Source: BMC Biol. 2026 Apr 11;24:121. doi: 10.1186/s12915-026-02593-9 (PMC13188829; doi:10.1186/s12915-026-02593-9)

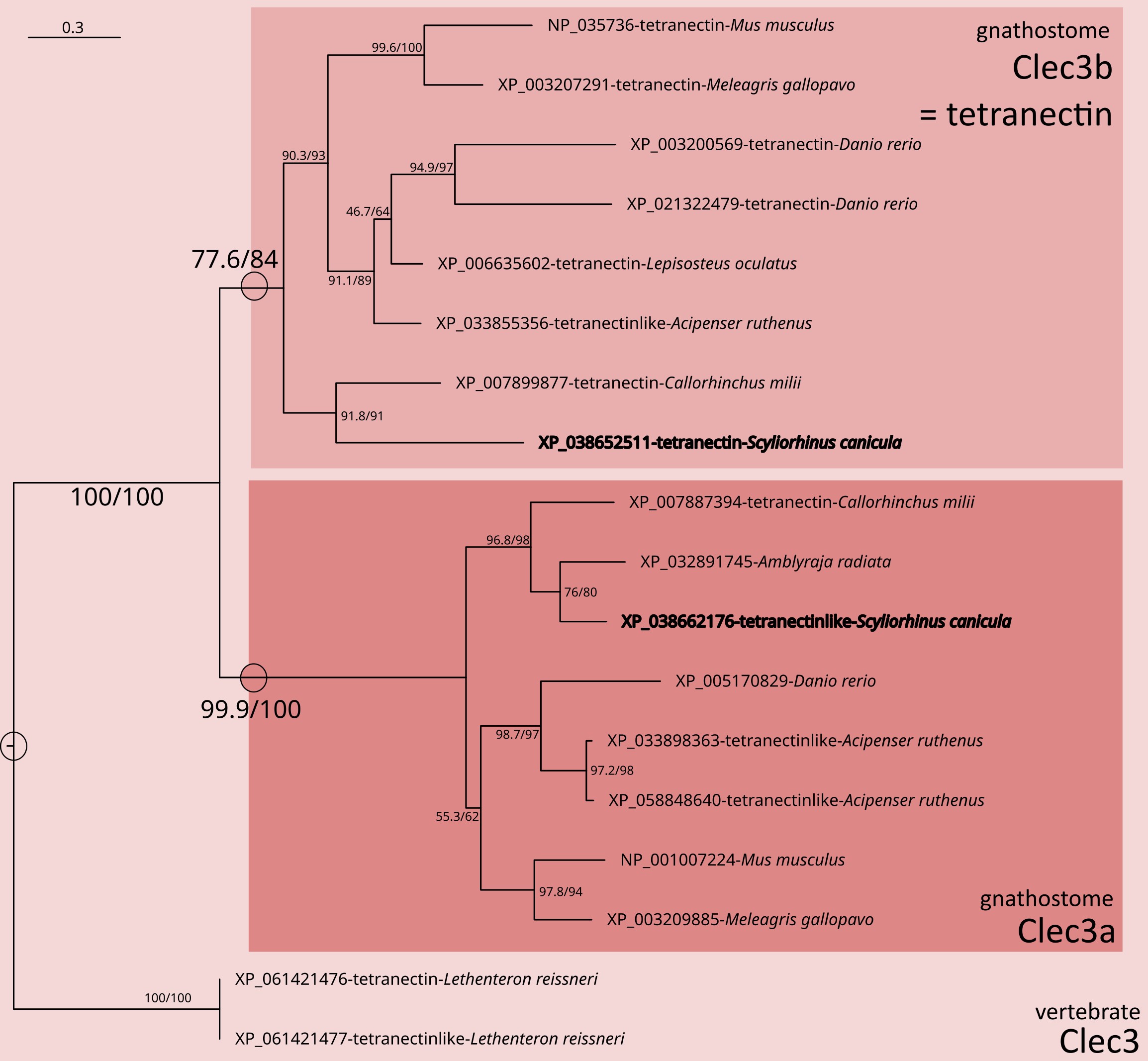

Supplement: Supplementary file 4 — Additional file 4. Figure S1. Phylogenetic relationships of Clec3a-related sequences in vertebrates obtained by maximum likelihood (best fit model according to BIC: Q.plant+I+G4; 18 sequences, 230 amino-acid positions) rooted by the lamprey Lethenteron reissneri closest sequences. Highlighted clades represent gnathostome orthology groups, SH-aLRT/UFBoot values for internal nodes are shown on each node. The small spotted catshark sequence to be identified is XP_038652511.1 in bold and belongs to the gnathostome Clec3a group of orthology. [file 12915_2026_2593_MOESM4_ESM.jpg]

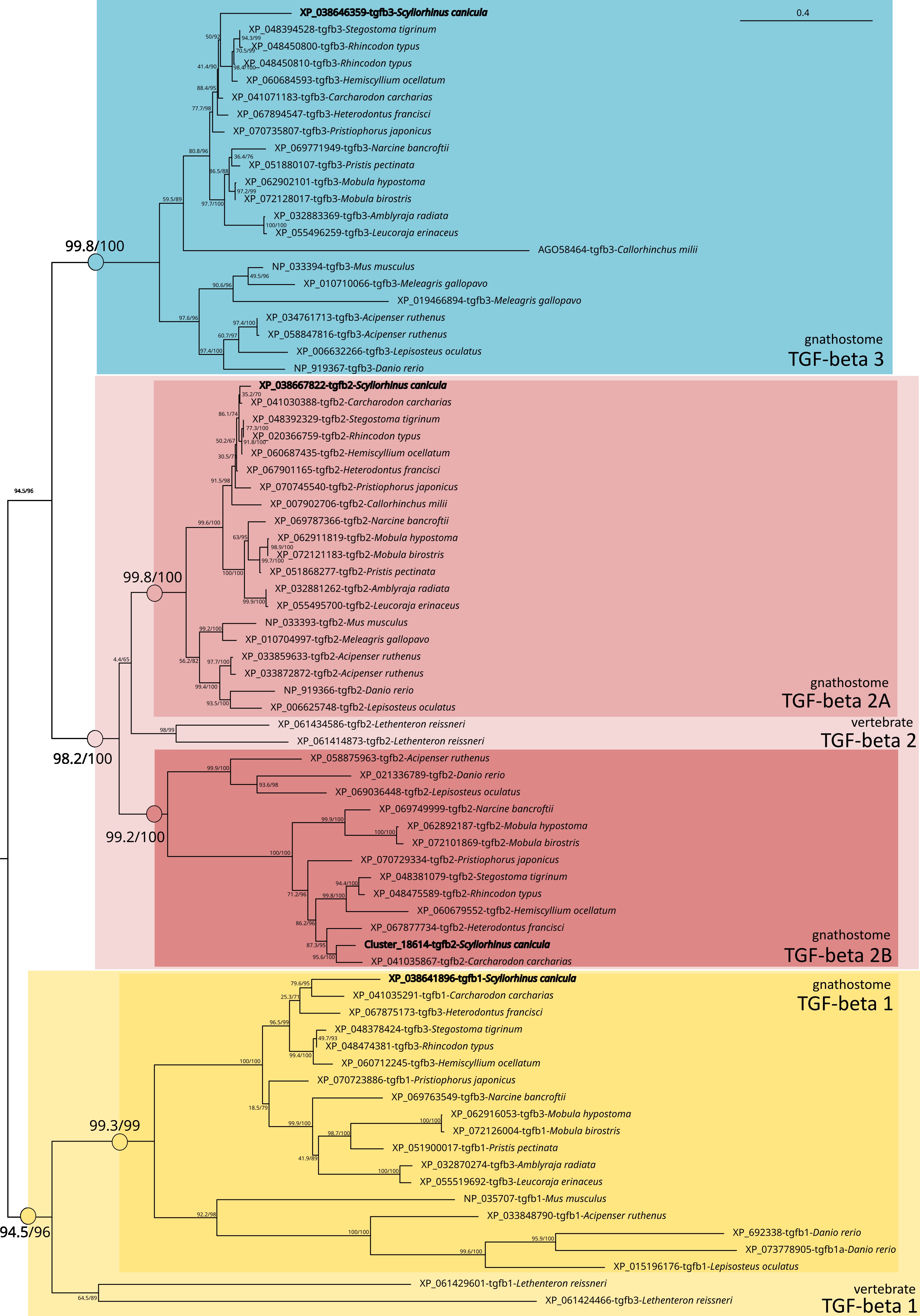

Supplement: Supplementary file 5 — Additional file 5. Figure S2. Phylogenetic relationships of Tgf-beta sequences in vertebrates obtained by maximum likelihood (best fit model according to BIC: JTT+I+G4; 77 sequences, 502 amino-acid positions) rooted by the Tgf-beta1 vertebrate clade as in [33]. Highlighted clades represent gnathostome orthology groups, SH-aLRT/UFBoot values for internal nodes are shown on each node. The small spotted catshark sequence to be identified is Cluster-18614 in bold and belongs to the gnathostome TGF-beta2B group of orthology (gene nomenclature follows [33]). [file 12915_2026_2593_MOESM5_ESM.jpg]

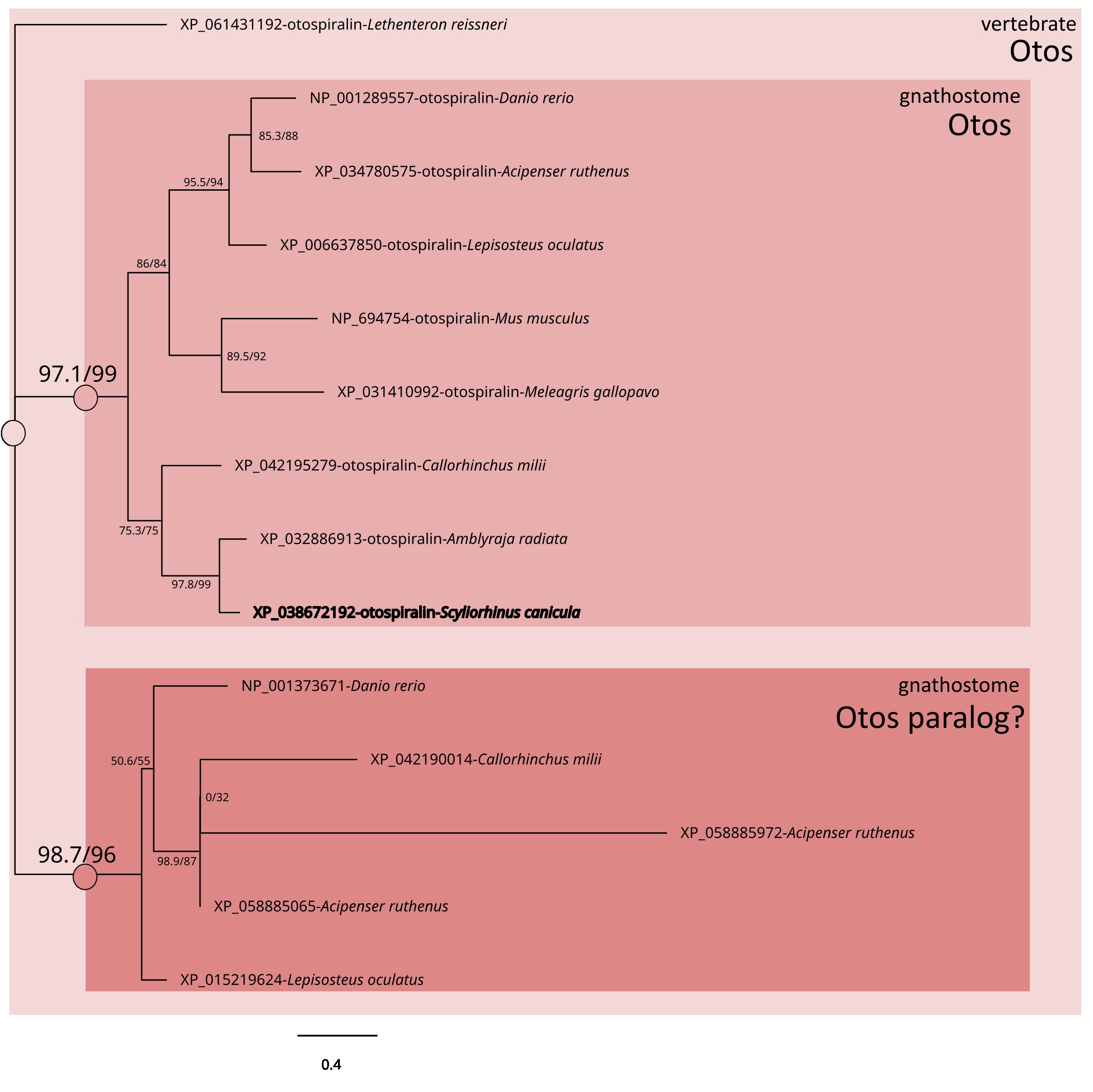

Supplement: Supplementary file 6 — Additional file 6. Figure S3. Phylogenetic relationships of Otos sequences in vertebrates obtained by maximum likelihood (best fit model according to BIC: JTT+G4; 14 sequences, 131 amino-acid positions) rooted by the lamprey Lethenteron reissneri closest sequence. Highlighted clades represent gnathostome orthology groups, SH-aLRT/UFBoot values for internal nodes are shown on each node. The small spotted catshark sequence to be identified is in bold and belongs to the gnathostome Otospiralin group of orthology. [file 12915_2026_2593_MOESM6_ESM.jpg]

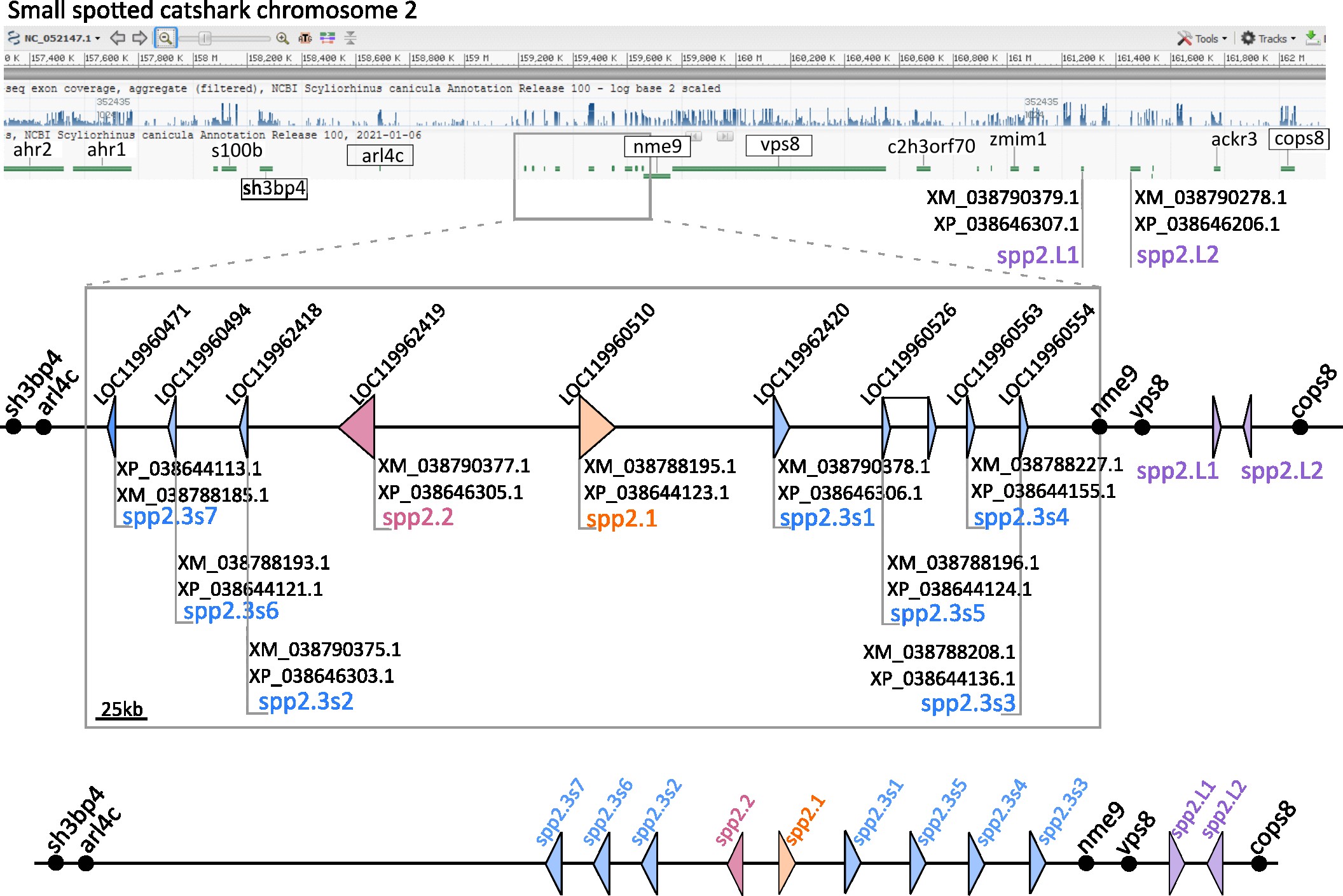

Supplement: Supplementary file 8 — Additional file 8. Figure S4. Scyliorhinus canicula spp2 gene cluster on chromosome 2. The colour code follows the one of clades in the phylogeny (see Figure 6). [file 12915_2026_2593_MOESM8_ESM.jpg]
